# Unveiling viral threats to temperate pome fruits: characterization, transmission, and sustainable management strategies

**DOI:** 10.3389/fmicb.2025.1560720

**Published:** 2025-03-19

**Authors:** Subaya Manzoor, Sajad Un Nabi, Aadil Ayaz, Bushra Rasool, Susheel K. Sharma, M. H. Chesti, Shugufta Parveen, M. K. Verma, M. A. Diab, Muhammad Fazle Rabbee

**Affiliations:** ^1^Plant Virology Laboratory, ICAR-Central Institute of Temperate Horticulture Srinagar, Jammu and Kashmir, India; ^2^Division of Plant Pathology, ICAR-Indian Agricultural Research Institute, New Delhi, India; ^3^Division of Soil Science and Agricultural Chemistry, SKUAST-K, Wadura, India; ^4^Apple Research Station, Pahnu Shopian, SKUAST-K, Shalimar, India; ^5^Department of Biotechnology, Yeungnam University, Gyeongbuk, Gyeongsan, Republic of Korea

**Keywords:** temperate, fruits, apple, pear, quince, virus, vector, detection

## Abstract

Apple (*Malus × domestica* Borkh.), pear (*Pyrus communis* L.), and quince (*Cydonia oblonga* Mill.) are widely cultivated fruit crops in temperate regions due to their desirable flavors and health benefits. However, their production is severely affected by various biotic stresses, with viral diseases being particularly significant challenge. These viral infections are of great economic importance, not only reduce tree vigor and yield but also compromise fruit quality and marketability. To date, more than 26 viruses and viroids have been identified as pathogens of these fruit trees. Many of these viral diseases persist as latent infections, causing permanent infections in these fruit trees. This review provides an overview of the viral pathogens affecting apple, pear, and quince, including their characterization, transmission modes, and the challenges they present for management. Emphasis is placed on accurate diagnosis and effective control strategies to mitigate the impact of these diseases in apple orchards.

## 1 Introduction

Temperate fruit crops belonging to the family *Rosaceae* include both pome and stone fruits. Pome fruits, such as apple *Malus domestica*, pear *Pyrus communis*, and quince *Cydonia oblonga*, are distinguished by their fleshy structures, in which the outer portion of the fruit is formed from the expanded floral parts and receptacle (Ali, 2023). The domesticated apple, pear, and quince are believed to have originated from the Caucasus Mountains, Western Asia, and Western China, respectively (Sabir et al., 2015). These trees are deciduous and require a winter dormancy period with exposure to cold temperatures to break dormancy and initiate spring growth. Pome fruits develop from spring blossoms and are typically harvested from late summer through autumn. Globally, apples and pears are major fruit crops, predominantly grown in countries such as China, the USA, Turkey, Poland, India, France, Italy, Germany, and Argentina, with China being the largest producer both in terms of yield and cultivated area (Chidembra, 2023).Regarding quince production, Turkey is the largest global producer, contributing 25% of total production, followed by China, Iran, Argentina, and Morocco, which together account for approximately 10% of global quince production (Postman, 2008). Apple is one of the most significant and widely cultivated fruit crops globally, particularly within the *Rosaceae* family (Tijero et al., 2021). Known as the “king of temperate fruits,” apples are highly regarded for their flavor and extensive global demand, making them a valuable export commodity. Apples are rich in essential vitamins and minerals, aid in weight loss, enhance cognitive health, and provide antioxidants, which can reduce the risk of metabolic syndrome and certain cancers. The pear (*Pyrus communis*), referred to as the “Gift of God” by the Greek poet Homer, is similarly valued for its health benefits. In Indian traditional medicine, it is known as “Amrithaphal,” meaning “fruit of immortality,” and has been traditionally used to treat gall bladder disorders, arthritis, colitis, and gout. Their regular consumption may help protect against colon cancer due to the fruit’s gritty fiber content, which binds to carcinogens and prevents their contact with the colon mucosa (Ghosh et al., 2015). The quince (*Cydonia oblonga* Mill.), an Asian fruit tree extensively cultivated in the Mediterranean and Central-Eastern Europe, is primarily used for producing jams, spirits, medicinal preparations, and flavorings. Additionally, quince is commonly employed as a rootstock for dwarf pear trees, which benefit from reduced vigor, earlier fruiting, and increased yield when grafted onto quince rootstock (Machado et al., 2014).

### 1.1 Virus and virus-like pathogens infecting apple, pear, and quince

Pome fruit cultivation is under threat from a variety of biotic stresses, including fungi, bacteria, viruses, viroids, and phytoplasmas, resulting in quantitative and qualitative yield losses (Nabi et al., 2020). Among these, viral diseases pose a serious global threat to apple, pear and quince production. These viral infections not only impede the tree growth and vitality, but also reduce marketable yields (Umer et al., 2019). Furthermore, viral infections are systemic, allowing them to be passed on to subsequent generations via propagating materials, ultimately deteriorating the plant’s overall health. The transmission of viruses and their vectors to new geographic areas is facilitated by the globalization of propagating material of these pome fruits, usually with unexpected effects. Because different viral pathosystems have different epidemiological features, there is no one-size-fits-all method for reducing the harmful impacts of viral diseases. Apple mosaic virus (ApMV), apple chlorotic leaf spot virus (ACLSV), apple stem grooving virus (ASGV), apple stem pitting virus (ASPV), prunus necrotic ringspot virus (PNRSV), apple necrotic mosaic virus (ApNMV), apple green crinkle associated virus (AGCaV), and viroids like apple scar skin viroid (ASSVd) and apple hammerhead viroid (AHVd) are among the at least 26 viruses and virus-like pathogens that can affect pome fruits in particular (Tables 1, 2), and their infections can cause significant financial losses. Most of the viruses are latent, but they establish permanent infections in fruit trees. Characterizing the specific viruses affecting pome fruits is crucial for understanding their transmission, host range, and impact on the crop. Integrated management approaches, including the use of virus-free planting material, strict quarantine measures, and targeted cultural practices, are essential in mitigating the spread of these viral diseases and ensuring the sustainability of pome fruit production in temperate regions.

**Table 1:** Major viruses infecting apple, pear and quince fruits, their host range, genome, and particle shape.

Family	Genus	Species	Genome	Particle	References
Bromoviridae	Ilarvirus	Apple mosaic virus	(+) ssRNA	Icosahedral	Grimová et al. (2016)
	Ilarvirus	apple necrotic mosaic virus	(+) ssRNA	Spherical	Noda et al. (2017)
	Ilarvirus	Apple ilarvirus 1			Wright et al. (2020)
	Ilarvirus	Apple ilarvirus 2			Xiao et al. (2022)
Luteoviridae	Luteovirus	Apple luteovirus 1	(+) ssRNA	Icosahedral	Liu et al. (2018)
	Luteovirus	Apple-associated luteovirus	(+) ssRNA	Icosahedral	Shen et al. (2018)
Fimoviridae	Emaravirus	Pear chlorotic leaf spot-associated virus	(−)ssRNA	Spherical	Liu et al. (2020)
Phenuiviridae	Coguvirus	Citrus virus A Citrus concave gum-associated virus	(+) ssRNA		Wang et al. (2022) and Khan et al. (2024a,b)
	Rubodvirus	Apple rubbery wood virus 1	(−)ssRNA		Rott et al. (2018)
	Rubodvirus	Apple rubbery wood virus 1	(−)ssRNA		Rott et al. (2018)
Secoviridae	Cheravirus	Cherry leaf roll virus	(+) ssRNA	Isometric	Woo et al. (2012)
	Nepovirus	Tomato ringspot virus	(+) ssRNA		Cummins and Gonsalves (1982)
	Nepovirus	Temperate fruit decay-associated virus	ssDNA		Basso et al. (2015)
Betaflexiviridae	Capillovirus	Apple stem grooving virus	(+) ssRNA	Filamentous	Yoshikawa et al. (1992)
	Foveavirus	Apple stem pitting virus	(+) ssRNA	Filamentous	Jelkmann (1994)
	Foveavirus	Apple green crinkle associated virus	(+) ssRNA	Filamentous	Morelli et al. (2017)
	Trichovirus	Apple chlorotic leaf spot virus	(+) ssRNA	Filamentous	German et al. (1990)

**Table 2:** Geographical distribution of viruses infecting pome fruit.

Virus name	Commonly affected hosts	Symptoms	Geographic distribution	References
Apple chlorotic leaf spot virus (ACLSV)	Apple, Pear, Quince	Leaf chlorosis, ring spots, stunted growth	Worldwide	Pedrelli et al. (2025)
Apple mosaic virus (ApMV)	Apple, Pear	Mosaic, chlorotic spots, leaf deformation	Worldwide	Suman et al. (2025)
Apple stem grooving virus (ASGV)	Apple, Pear, Quince	Grooves on stems, stunting, yield reduction	Worldwide	Mosquera-Yuqui et al. (2025)
Apple stem pitting virus (ASPV)	Apple, Pear, Quince	Pitting on stems, decline of trees	Worldwide	Rithesh et al. (2025)
Apple scar skin viroid (ASSVd)	Apple	Small, scarred, and cracked fruit	Asia, Europe, North America	Mosquera-Yuqui et al. (2025)
Apple dimple fruit viroid (ADFVd)	Apple	Dimpled fruit, size reduction	Asia, Europe	Chambers et al. (2025)
Tomato ringspot virus (ToRSV)	Apple, Pear	Ringspots, necrotic streaks, reduced vigor	North America	Obaid and Adhab (2025).
Cherry rasp leaf virus (CRLV)	Apple, Pear	Leaf curling, deformation, stunting	North America	Shevchenko et al. (2025).
Pear blister canker viroid (PBCVd)	Pear	Cankers, necrosis, reduced fruit production	Europe	Aslam et al. (2025).
Pear vein yellows virus (PeVYV)	Pear	Vein yellowing, mottling, delayed leaf fall	Europe	Balendres et al. (2025)
Asian pear virus 1 (APV1)	Pear	Leaf chlorosis, reduced growth	Asia	Zhang et al. (2025)
Apple rubbery wood virus (ARWV)	Apple	Softening and loss of rigidity in wood	Europe, North America	Khan et al. (2024a,b)
Pear latent virus (PeLV)	Pear	Often asymptomatic, mild chlorosis in leaves	Europe, Asia	FontdevilaPareta et al. (2024)
Apple green crinkle-associated virus (AGCaV)	Apple	Fruit deformation, green crinkle symptoms	Europe, North America	Chofong et al. (2024)
Apple latent spherical virus (ALSV)	Apple, Pear	Often symptomless, potential yield loss	Asia, Europe	Vats et al. (2024)
Apple ringspot virus (ApRSV)	Apple, Pear	Ringspots on leaves, chlorosis, stunting	Asia	Orhan and Yeşil (2025)
Apple chat fruit viroid (ACFVd)	Apple	Fruit deformation, yield loss	Asia	Vadamalai et al. (2024)
Apricot latent virus (ApLV)	Apple, Pear	Often symptomless, potential fruit yield loss	Europe	Jevremović and Paunović (2024)
Little cherry virus 1 (LChV1)	Apple	Reduced fruit size, poor fruit quality	North America, Europe	Jevremović et al. (2025)
Little cherry virus 2 (LChV2)	Apple	Severe fruit deformity, delayed ripening	North America	Jevremović et al. (2025)

## 2 Major viral diseases of apple, pear, and quince

### 2.1 Mosaic disease

Mosaic disease is prevalent across apple, pear, and quince-growing regions, causing significant qualitative and quantitative damage to the crop. The infection manifests as pale to bright cream-colored spots on newly expanded spring leaves, with some cultivars displaying pronounced white banding patterns along the major veins. Common symptoms include irregular pale yellow to bright cream spots or bands along the main veins of spring leaves, along with characteristic yellow line patterns, bright yellow blotches, rings, and vein clearing. Initially, the apple mosaic virus (ApMV) was thought to be the only cause of this disease. But recent investigations have revealed that the main cause of mosaic disease in apples, pear and quince particularly in China, Korea, Japan, and India, is a novel virus designated apple necrotic mosaic virus (ApNMV) (Noda et al., 2017; Nabi et al., 2020). Mosaic disease can cause yield losses of 30–50%; losses for the Golden Delicious cultivar can reach 46% (Cembali et al., 2003). ApMV and ApNMV are both members of the *Bromoviridae* family, namely the genus *Ilarvirus*. The genus *Ilarvirus* contains viruses with icosahedral particles and tripartite genomes. The RNA 1, RNA 2, RNA 3, and a subgenomic RNA 4 comprise the well-characterized ApMV genome (Roossinck et al., 2005). Three quasi-spherical or somewhat pleomorphic particles with dimensions ranging from 25 to 29 nm make up an ApMV virion. The positive-sense, single-stranded RNA virus known as ApMV requires the presence of all of its genomic components in the host plant in order to infect it. Plant RNA viruses are known to have methyltransferase-like and helicase-like (MET/HEL) domains in their big polypeptide encoded by RNA 1, the longest genomic segment at 3,476 nucleotides (nt). The sole big open reading frame (ORF) of RNA 2, a 2,979 nt long molecule, has sequence similarity with motifs present in the majority of viral RNA-dependent RNA polymerases (RDRPs). RNA 3, which is 2,056 nt long, contains two ORFs: the first encodes a movement protein (MP) that facilitates cell-to-cell movement and is directly translated from RNA 3, while the second ORF encodes the coat protein (CP), which is translated from sub-genomic RNA 4 derived from RNA 3. Similarly, ApNMV is a positive-sense, single-stranded RNA virus. Its genome consists of three RNAs RNA 1, RNA 2, and RNA 3, with sizes ranging from 3,378 to 3,380 nt (RNA 1), 2,778 to 2,786 nt (RNA 2), and 1,909 to 1,955 nt (RNA 3), respectively (Nabi et al., 2022a,b). The complete genome of ApNMV has been sequenced and confirmed, showing consistency with viruses in *Ilarvirus* subgroup 3. RNA 1 encodes the MET/HEL protein, which is 1,056 amino acids (aa) long. RNA 2 encodes an RNA polymerase protein that is 855–867 amino acids long and features the highly conserved GDD motif, typical of *Ilarviruses*, located at positions 627–630 (or 626–629). RNA 3 encodes two proteins: the movement protein (280–281 amino acids) and the coat protein (219 amino acids), with the coat protein likely produced from sub-genomic RNA 4 (Xing et al., 2018). The intergenic region (IR) between the movement protein and coat protein genes is believed to be crucial, potentially serving as a promoter for the transcription of RNA 4 from RNA 3 by the viral replicase (Xing et al., 2018). Both viruses can be transmitted through grafting, and currently, no insect vectors have been identified (Manzoor et al., 2023). Cucumber mosaic virus (CMV) has been implicated in causing mosaic disease in both apple (Hu et al., 2016) and pear (Lee et al., 2018). As the type species of the genus *Cucumovirus* within the family *Bromoviridae*, CMV is characterized by three spherical particles, each measuring approximately 28 nm in diameter. Its genome comprises three single-stranded, positive-sense RNA molecules: RNA 1 (~3,350 nucleotides), RNA 2 (~3,050 nucleotides), and RNA 3 (~2,200 nucleotides). Each RNA is encapsulated in its own protein coat, resulting in a mature viral particle composed of these three distinct components. Additionally, RNA 3 can associate with a fourth RNA strand, referred to as RNA 4 (~1,030 nucleotides), which encodes the coat protein. This coat protein is synthesized from a subgenomic RNA generated during replication, with RNA 3 housing the gene but relying on RNA 4 for protein expression. The replication and translation processes of CMV involve specific functions for each RNA. RNA 1 encodes the essential replication protein 1a, while RNA 2 produces two proteins, 2a and 2b, with 2a playing a critical role in viral genome replication. RNA 3 encodes proteins 3a, known as the movement protein (MP), and the coat protein. The 3a protein facilitates intercellular movement of the virus, and mutations in this protein can influence movement efficiency in a host-dependent manner. The coat protein, found in viral particles, is essential for cell-to-cell movement and serves as the primary factor for aphid-mediated transmission, although its role in movement is likely indirect (Zitter and Murphy, 2009).

### 2.2 Rubbery wood disease

Rubbery wood is caused by the apple rubbery wood virus (also known as apple rubodvirus), named after the prefix “Rub-” in “Rubbery” and the suffix “-od” in “wood.” The virus primarily infects apple and pear trees, causing an unusual flexibility in twigs and smaller branches due to reduced lignification of xylem vessels and fibers, leading to a lack of rigidity. Apple rubodvirus 1 (ARWV1) and apple rubodvirus 2 (ARWV2) are classified under the genus *Rubodvirus* within the newly established family *Phenuiviridae*, part of the order *Bunyavirales*. The virus spreads through grafting and budding, but no natural vector has been identified. It can significantly reduce fruit yield, with losses ranging from 10 to 30%.The genomes of both ARWV-1 and ARWV-2 are helical, tripartite, negative-sense single-stranded RNA (-ssRNA). Each genomic RNA segment has one open reading frame (ORF) and a complementary RNA (cRNA). The three RNA segments, designated as large (L), medium (M), and small (S), encode an RNA-dependent RNA polymerase (RdRp), a movement protein (MP), and a nucleocapsid protein (NP), respectively. The primary difference between ARWV-1 and ARWV-2 lies in their encoded proteins, with sequence identities ranging from 59% for RdRp proteins to approximately 66–68% for NP proteins (Rott et al., 2018).

### 2.3 Rapiddecline or sudden decline

The disease is caused by apple associated *Luteovirus* (AaLV) or *Luteovirus* sociomali, belonging to family *Tombusviridae*, genus *Luteovirus* and is considered a significant threat to apple orchards and the apple industry. In severe cases, the virus can lead to tree death and orchard losses, which can have a substantial economic impact on apple growers and the apple industry. The virus particles of AaLV are roughly spherical or icosahedral in shape, with a regular geometric structure. Due to the rapid or sudden death of apple trees caused by AaLV, it is known as rapid apple decline. The disease starts as leaf discoloration and trunk cracking, and tree vigor is severely affected, eventually resulting in rapid decline. The complete genome sequence of AaLV comprises 5,890 nt and contains eight open reading frames (ORFs), in a very similar arrangement that is typical of members of the genus *Luteovirus* (Liu et al., 2018).

### 2.4 Union necrosis and decline

Union necrosis and decline is caused by tomato ring spot virus (ToRSV). In infected apple pear and quince trees budbreak may be delayed, leaves might be small and sparse, and pale green, shoot growth is reduced and causes ringspots. Terminal shoot growth is reduced, with shortened internodes. Infected trees flower heavily and set large numbers of small, highly colored fruit. Leaf discoloration and leaf drop occur prematurely in infected trees. Affected trees often produce large numbers of sprouts from the rootstock. Swelling may occur above the graft union. Partial to complete separation of the graft union is common on severely affected trees; sometimes the top breaks off at the union in strong winds. The virus belongs to family *Secoviridae* and orders *Picornavirales* and is non-enveloped linear (Teliz et al, 1966). The genome is (+) sense RNA which contains a VPg at the 5′ termini and a poly(A) tail at the 3′ termini. The TomRSV RNA1 is 8,214 nucleotides in length, excluding the 3′ poly(A) tail, and contains a single long open reading frame (ORF) of 6,591 nucleotides beginning at the first AUG codon at nucleotide position 78. This ORF accounts for 80% of the RNA1 sequence and would give rise to a polyprotein with a predicted molecular mass of 244 kDa (Rott et al., 1995).

### 2.5 Green crinkle disease

Green crinkle disease, also known as kikei-ka disease in Japan has significant economic consequences for apple and pear orchards. Infected trees may experience stunted growth, reduced vigor, and increased susceptibility to other diseases and stressors. The symptoms are characterized by ring-shaped rust on the fruits and puckering on leaves, wart-like swellings some of which may be covered with rough russet transmitted through grafts (Li et al., 2022). It is caused by apple green crinkle associated virus (AGCaV) which belongs to family *Betaflexiviridae* and genus *Foveavirus* that has been associated with apple green crinkle. Two strains of this virus have been described, one associated with apple green crinkle, while the second was found in quince (*Cydonia oblonga*) trees expressing quince fruit deformation or quince sooty ringspot disease symptoms (Morelli et al., 2017). The genome of the isolate AGCaV-CYD is ssRNA consisting of 9,266 nt, with five ORFs and four noncoding regions (NCRs).

### 2.6 Flat apple disease

Flat apple symptoms are most severe on “Red Delicious” and related cultivars. The length of the fruit is significantly reduced and the stem cavity becomes very shallow. At first, only few fruits become symptomatic on lower limbs, but eventually, the entire tree gets involved. Apple flat apple disease is caused by cherry rasp leaf virus (CRLV). Many weed species that inhabit the orchard floor are hosts to the virus. Cherry rasp leaf virus is a plant pathogenic virus of the order *Picornavirales*, family *Secoviridae*, and genus *Cheravirus*. The CRLV is transmitted usually by nematode (*Xiphinema americanum*), mechanical inoculation, grafting, or seed (10–20%). Since, the virus is nematode transmitted, it spreads slowly in the orchard. The length RNA1 is 6.8–7.1 kb and RNA2 is 3.2–3.7 kb-long. Each genomic RNA encodes a polyprotein (RNA1: P1; RNA2: P2) that is cleaved by the RNA1-encoded 3C proteinase to generate the functional non-structural and structural proteins (CRLV) encode three capsid proteins (James et al., 2001).

### 2.7 Citrus concave gum-associated virus

Citrus concave gum-associated virus (CCGaV) was reported in citrus trees in Italy, CCGaV belongs to the genus *Coguvirus* within the family *Phenuiviridae*. It possesses a bipartite genome comprising one negative-strand RNA (RNA1) and one ambisense RNA (RNA2). RNA1 encodes the RNA-dependent RNA polymerase (RdRp), while RNA2 encodes the movement protein (MP) and nucleocapsid protein (NP) (Navarro et al., 2018). In recent years, CCGaV was also been detected in apple trees in China, Italy, and the USA (Minutolo et al., 2021). This virus was also reported from apple crop in India (Khan et al., 2024a,b). Citrus concave gum-associated virus (CCGaV) infection in apple trees has not been extensively studied, but based on its effects in other hosts, it is likely associated with symptoms such as reduced tree vigor, leaf chlorosis, and mottling. Infected trees may exhibit stunted growth, branch deformation, and possible bark abnormalities, including cracking or concave depressions similar to those observed in citrus. Additionally, fruit development may be affected, leading to smaller, lower-quality apples with irregular ripening. Some infections may also cause a rubbery or spongy wood texture, as seen in other apple viruses. Nevertheless, further research is needed to fully understand the impact of CCGaV on apple orchards.

### 2.8 Latent infections

#### 2.8.1 Apple latent spherical virus

The Apple Latent Spherical Virus (ALSV) is a significant viral pathogen affecting apple orchards and related fruit crops, leading to substantial economic losses. This virus compromises fruit quality and reduces marketable yield, thereby impacting overall agricultural productivity and profitability. The ALSV belongs to family *Secoviridae* and is transmitted through pollen grains. The virus had two ssRNA species (RNA1 and RNA2) and three capsid proteins (Vp25, Vp24, and Vp20). The complete nucleotide sequences of RNA1 and RNA2 determined to be 6,815 and 3,384 nt excluding the 3′ poly(A) tail, respectively. RNA1 contains two partially overlapping ORFs encoding polypeptides of molecular mass 23 kDa (“23 K”; ORF1) and 235 kDa (“235 K”; ORF2); RNA2 has a single ORF encoding a polypeptide of 108 kDa (Li et al., 2000).

#### 2.8.2 Apple chlorotic leaf spot virus

Apple chlorotic leaf spot virus (ACLSV) causes significant economic losses in apple, pear and quince orchards. The disease reduces the photosynthetic capacity of the affected leaves, leading to decreased fruit quality and yield. In severe cases, it results in premature fruit drop. Infection is generally latent in apple crop but sometimes symptoms include, bark splitting or severe fruit deformations, chlorotic rings, russet rings on fruits, graft incompatibility, and bud necrosis. It leads to 40% yield loss in pear. Apple chlorotic leaf spot virus belongs to the genus *Trichovirus* and family *Betaflexiviridae*. The ACLSV is flexuous, filamentous, positive sense single stranded RNA virus with a genome ranging between 7,545 and 7,555 nt in length that comprises three open reading frames (ORFs). ORF1 encodes a large replication-related protein containing polymerase (RdRp), nucleotide binding helicase, and methyltransferase domains, ORF2 encodes a movement protein (MP), and ORF3 encoding the coat protein (CP) (Nabi et al., 2022a,b).

#### 2.8.3 Apple stem pitting virus

The economic importance of apple stem pitting virus lies in its detrimental effects on apple orchards and fruit production. It leads to reduced fruit yield in affected trees. The virus affects trees vascular system and impairs the movement of nutrients and water, impacting fruit size and development. ASPV is member of *Foveavirus* species and genus from *Betaflexiviridae* family. It possesses a single stranded positive RNA (+ssRNA) genome comprising of approximately 9,300 nucleotides (nts), encoding five open reading frames (ORFs, ORF1-ORF5) as well as the 5′ untranslated region (UTR) and 3′ UTR. ORF1 encodes the viral RNA-dependent RNA polymerase (RdRP), ORF2-ORF4 encode triple gene block proteins (TGBp1-TGBp3) and ORF5 encodes the viral coat (capsid) protein (CP). ASPV consists of flexuous, filamentous particles, approximately 800 nm in length and 12–15 nm in width, with helical symmetry. ASPV infects several plant species and causes a wide range of symptoms from symptomless to xylem pits, epinasty, decline, vein yellowing, leaf red mottling, pear necrotic spot or fruit stony pits depending on the plant species, the cultivar and the viral strain/isolate (Jelkmann, 1994). ASPV is transmitted mechanically by grafting and through infected propagative material. As of now, there is no known vector causing its transmission.

#### 2.8.4 Apple stem grooving virus

Apple stem grooving virus (ASGV) is a plant virus that primarily infects apple, pear and quince trees and can have significant economic implications for the pome fruit industry. As the virus affects the tree’s vascular system, it can impair the movement of nutrients and water, impacting fruit development and size. In pear it leads to black necrotic leaf spot disease symptoms (Cho et al., 2010). The virus belongs to genus *Capillovirus*, subfamily *Trivirinae*, family *Betaflexiviridae,* and order T*ymovirales*. The virus is latent on apple cultivars, however sometimes causes stem grooving, brown line, and graft union abnormalities only when an infected cultivar is grafted on a sensitive rootstock such as *Malus pumila*. Virginia, ASGV has filamentous virions, each composed of a positive-sense, single-stranded RNA genome of about 6,500 nucleotides excluding the non-stranslated 5′ and the 3′ polyadenylated tail. The virus is comprised of two ORFs: ORF1 encoding a polyprotein containing motifs of methyltransferase, papain-like protease, the nucleotide triphosphate-binding helicase, the RNA polymerase (RdRp), and the coat protein (CP); and ORF2 that encodes the movement protein (MP). The virus is seed and mechanically transmitted and no natural vectors have been identified to date (Nabi et al., 2022a,b).

#### 2.8.5 Temperate fruit decay associated virus

Temperate fruit decay-associated virus (TFDaV) is known to systemically infect apple and pear trees, leading to reduced growth rates. The relatively brief observation period of 180 days for these perennial woody hosts may explain the absence of visible foliar symptoms, as such hosts typically undergo latent infection periods ranging from 2 to 3 years (Al Rwahnih et al., 2009). Furthermore, the lack of foliar symptoms associated with TFDaV infection may be attributable to co-infections with other viruses or virus-like agents present in the original trees from which TFDaV was isolated. The rolling circle amplification (RCA) method has demonstrated efficacy in detecting single-stranded DNA (ssDNA) viruses, facilitating the identification of novel ssDNA viruses across various plant species through its unbiased sequencing capabilities (Inoue-Nagata et al., 2004). While RCA effectively detected TFDaV in field plants, the observed viral incidence remains low, and the survey was limited in scope. Therefore, further research is crucial to accurately assess the incidence of TFDaV, its potential economic impact, and the possibility of additional host species. TFDaV, classified within the genus *Temfrudevirus* of the family *Amesviridae*, features a monopartite circular ssDNA genome approximately 3.4 kb in length. This virus exhibits similarities to members of the *Circoviridae*, *Nanoviridae*, and *Geminiviridae* families, characterized by a conserved non anucleotide sequence, TAGTTATTAC, located within an intergenic region that includes a short palindromic sequence capable of forming a stem-loop structure (Jevremović and Paunović, 2024). Additionally, the intergenic region contains three iteron-like tandem repeat sequences, which likely function as binding sites for the replication (Rep) protein.

#### 2.8.6 Pear chlorotic leaf spot-associated virus

The pear chlorotic leaf spot-associated virus (PCLSaV) is the cause of the recently discovered disease known as pear chlorotic leaf spot (PCLS) (Liu et al., 2020). Semi-transparent chlorotic patches, necrotic spots on the stem bark, and ringspots on immature leaves are some of the symptoms. PCLSaV is a member of the *Fimoviridae* family and genus *Emaravirus*. Its genome is comprised of up of five segments of negative-sense RNA (Wang et al., 2022). A single open reading frame (ORF) and two complementary 13-nucleotide lengths at the 5′ and 3′ termini are present in each RNA segment. With a calculated molecular weight (MW) of 268 kDa, RNA1, a 7,100 nt RNA-dependent RNA polymerase, is encoded by this 7,100 nt RNA. A glycoprotein precursor (P2) with a molecular weight of 69.3 kDa is encoded by RNA2, which is 2,045 nt long. The 1,296 nt long RNA3 encodes the nucleocapsid protein (NP, P3), which has a 30 kDa projected molecular weight. At 1,543 nucleotides, RNA 4 codes for a protein (P4) with a projected molecular weight (MW) of 37 kDa and 321 amino acids. A protein (P5) with a molecular mass of 32.4 kDa and 282 amino acids.

#### 2.8.7 Citrus virus A

Citrus virus A (CiVA) presents symptoms of pear vein yellows on young leaves. The genome of CiVA is bipartite, consisting of one ambisense and one negative-strand RNA. It is closely linked to the type species of the recently recognized genus *Coguvirus* in the family *Phenuiviridae,* order *Bunyavirales*, which is citrus concave gum-associated virus (CCGaV) (Liu et al., 2020). While RNA2 encodes a nucleocapsid protein and a movement protein (ORF2a, viral strand), full-length RNA1 (6,690 nt) encodes the hypothesized RNA-dependent RNA polymerase (Svanella-Dumas et al., 2019).

## 3 Transmission viruses infecting pome fruits

Pome viruses can be transmitted in various ways, and it is important for orchard managers to understand their transmission methods to prevent the spread of these viruses. Apple viruses are typically spread through various means: grafting, budding, mechanical, insects and nematodes (Grimová et al., 2016; Li et al., 2022; Nabi et al., 2024). One of the most common ways for apple, pear and quince viruses to spread are through propagation, grafting, and top working, when infected scion buds are carelessly grafted onto healthy rootstock or rootstock that is already infected with the virus. This causes virus transmission between the infected scion bud and the rootstock, or vice versa, resulting in tree infection and decline. Nematodes are tiny worm-like organisms can also spread certain viruses by feeding on the roots of infected trees and then spreading the virus to other trees. Transfer of a viruses from an infected plant to a healthy one through physical means, such as pruning tools or hands also occur in some apple, pear and quince viruses like apple stem grooving virus. The mode of transmission of pome viruses are shown in Table 3.

**Table 3:** Mode of transmission of the major viruses infecting apple, pear, and quince fruits.

S. no.	Mode of transmission	Name of virus	References
1	Grafting/budding	Apple mosaic virus	Grimová et al. (2016), Noda et al. (2017), Morelli et al. (2017), Liu et al. (2018), and Rott et al. (2018)
		Apple necrotic mosaic virus	
		Apple chlorotic leaf spot	
		Apple green crinkle associated virus	
		Apple rubodo virus	
		Apple associated luteovirus	
2	Nematode	Cherry rasp leaf virus	James et al. (2001) and Rott et al. (1995)
	(*Xiphinema Americana*)	Tomato ring spot virus	
3	Mechanical	Apple stem grooving virus	Nabi et al. (2022a,b)
		Apple stem pitting virus	
4	Pollen	Apple latent spherical virus	Li et al. (2000)
5	Thrips	Temperate fruit decay associated virus	Al Rwahnih et al. (2009)
7	Seeds	Apple stem grooving virus	Li et al. (2022)
		Apple stem pitting virus	
		Apple chlorotic leaf spot	
		Apple rubodo virus	
		Apple chlorotic leaf spot	
		Apple green crinkle associated virus	
8	Aphids	Cucumber mosaic virus	Zitter and Murphy (2009)

## 4 Reliable and sensitive detection methods of pome fruit viruses

Accurate diagnosis of diseases and their causal agents is essential for effective disease management (Piombo et al., 2021). Consequently, diagnosis plays a pivotal role in managing these viruses. The primary methods used for detecting and identifying viruses in apple, pear, and quince include biological assays and the virus’s intrinsic properties, utilizing both serological and molecular assays (Barba et al., 2015).

### 4.1 Detection based on biological properties or biological methods

Biological diagnosis, also known as biological indexing, has been a key method for assessing plant health and identifying or characterizing specific viruses. This technique involves observing symptoms on natural hosts or susceptible indicator plants, relying on the interaction between the virus and its host. In woody indicator plants, grafting or chip budding is commonly used (Hu et al., 2019). Several fruit tree genotypes, such as *Malus platycarpa*, *Malus micromalus*, and *Malu spumila* cultivars like “Virginia Crab” or “Spy227,” have been employed for detecting apple viruses (Jevremović and Paunović, 2024). The development of symptoms by ApMV in test plants such as; Golden Delicious (Mink, 1998), Crab Apple (Hu et al., 2019), and *Malus silvestris* cultivars like, Lord Lambourne, and, Jonathan (Fulton, 1972), provides initial evidence of the virus’s presence in the field or laboratory. However, this method is labor-intensive, time-consuming, and prone to reliability issues and subjective interpretation, making it less practical for modern virus diagnostics.

### 4.2 Detection based on viral proteins/serological methods

Methods are generally called as serological or immunoassays which use surface properties of viral proteins for detection of viruses. Mostly coat protein (CP) involved in making of capsid of a virus is used for detection (Table 4). The immunological assays like enzyme linked immunosorbant assay (ELISA) stands out as one of the most adaptable immunodiagnostic techniques. It is dependent on the precise interactions between proteins and antibodies, typically focusing on the desired virus’s capsid protein. The ApMV, ASPV, ASGV and ACLSV has been detected using Double antibody sandwich-ELISA both in India as well as elsewhere in the world (Nabi and Baranwal, 2020).

**Table 4:** Detection of apple, pear, and quinces viruses.

S.no	Virus	Detection method	References
01	ApMV	Visual symptoms	Posnette and Cropley (1956)
		ELISA	Plopa and Preda (2013)
		RT-PCR	Nabi et al. (2020)
		Next generation sequencing (NGS)	Khan et al. (2024a,b)
		LAMP	Wani et al. (2023)
		Multiplex-PCR	Nabi et al. (2022a,b)
		DAS-ELISA	Nabi et al. (2020)
02	ApNMV	RT-PCR	Nabi et al. (2020)
		CRISPR/Cas12a	Jiao et al. (2021)
		RT-RPA-LFD	Jiao et al. (2021)
		Multiplex-PCR	Nabi et al. (2022a,b)
		RT-PCR	Khan et al. (2024a,b)
03	ARWV-1	Next generation sequencing (NGS)	Khan et al. (2024a,b)
		RT-PCR	Khan et al. (2024a,b)
04	CiVA	Next generationsequencing (NGS)	Khan et al. (2024a,b)
		RT-PCR	Khan et al. (2024a,b)
04	ASPV	Next generation sequencing (NGS)	Khan et al. (2024a,b)
		CRISPR/Cas12a	Jiao et al. (2021)
		RT-RPA-LFD	Jiao et al. (2021)
		Multiplex-PCR	Nabi et al. (2022a,b)
		RT-PCR	Khan et al. (2024a,b)
		DAS-ELISA	Nabi et al. (2020)
05	ASGV	Next generation sequencing (NGS)	Khan et al. (2024a,b)
		CRISPR/Cas12a	Jiao et al. (2021)
		RT-RPA-LFD	Jiao et al. (2021)
		Multiplex-PCR	Nabi et al. (2022a,b)
		RT-PCR	Khan et al. (2024a,b)
		DAS-ELISA	Nabi et al. (2020)
06	ACLSV	Next generation sequencing (NGS)	Khan et al. (2024a,b)
		CRISPR/Cas12a	Jiao et al. (2021)
		RT-RPA-LFD	Jiao et al. (2021)
		Multiplex-PCR	Nabi et al. (2022a,b)
		RT-PCR	Khan et al. (2024a,b)
		DAS-ELISA	Nabi et al. (2020)

### 4.3 Detection based on viral nucleic acid/via molecular methods

The most important techniques among these are Polymerase Chain Reaction (PCR), Reverse Transcriptase-PCR, Multiplex PCR and Quantitative PCR. Comparing nucleic acid-based detection methods to biological or sero-diagnostic methods reveals clear advantages. Nucleic acid-based detection techniques are the most promising method for detection because many viruses show low immunogenicity. Comparing these procedures to serological techniques reveals how much more effective, sensitive, and specific they are. Consequently, they are well-suited for the routine detection of apple viruses and have found widespread application in molecular detection endeavors. With the development and application of PCR (Polymerase Chain Reaction), a significant advancement in the detection, discovery, and characterization of viruses was made. Reverse transcriptase (RT) enzyme is used to synthesize a first-strand cDNA in the case of viruses with RNA genomes. The heat-stable polymerase enzyme is then used to amplify this cDNA *in vitro*. Sequencing is typically needed to confirm the precise identity of the viral portion of the genome that has been amplified through PCR or RT-PCR. RT-PCR was used for detecting the viruses ARWV-1, ApNMV, CiVA, ASPV, ASGV, ACLSV, and ASGV-Shimla in pear (Khan et al., 2024a,b). The major characterized viruses apple necrotic mosaic virus (ApMV), apple stem pitting virus (ASPV), apple chlorotic leafspot virus (ACLSV), and apple mosaic virus (ApMV) have all been detected in infected trees using multiplex-PCR (Nabi et al., 2022a,b). Multiplex-PCR has been used to simultaneously detect several well-known viruses in diseased trees, including Apple, like ApMV, ApNMV, ASPV, ACLSV (Nabi et al., 2022a,b). With this method, multiple viruses can be thoroughly and effectively diagnosed in a single assay.

### 4.4 Isothermal amplification

Isothermal amplification methods were a breakthrough in the field of molecular diagnostics, eliminating the need for a thermocycler to amplify nucleic acid. Being cheaper and simpler to perform, they can be easily used for field detection of viruses. In plant virus diagnostics, reverse transcriptase loop-mediated isothermal amplification (RT-LAMP) and recombinase polymerase amplification (RPA) have been prominently used due to their broad range of application and rapidity (Tan et al., 2022). The development of RT-LAMP as a substitute technique for PCR that can effectively amplify very low copies of target DNA in a remarkable short time has resulted as a result (Waliullah et al., 2022). Three sets of primers used in LAMP-internal, external, and loop-are created to complement six distinct regions of the template DNA. The reaction makes use of the high strand-displacement activity Bst DNA polymerase. The ability of LAMP to operate at a fixed temperature range of 60–65°C, obviating the need for repeated denaturation steps, is one of its distinguishing characteristics. The entire procedure usually only takes 30–60 min. The CP (coat protein) gene of the (ApMV) has been used for development of RT-LAMP (Wani et al., 2023). The most current isothermal technology, primer-recombinase amplification (RPA), has a lot of potential for the quick on-site detection of plant viruses and is very stable and reliable for diagnosing apple stem grooving virus (ASGV). In China, RT-RPA is utilized to identify different viruses that impact apple production in order to fight apple necrotic mosaic virus (ApNMV), apple stem pitting virus (ASPV), apple stem grooving virus (ASGV), apple chlorotic leaf spot virus (ACLSV) (Jiao et al., 2021).

### 4.5 Next-generation sequencing

Next-generation sequencing (NGS), also known as deep sequencing or high-throughput sequencing, involves sequencing the total nucleic acid content in symptomatic and asymptomatic samples to identify pathogens using bioinformatics tools. The NGS is a sequence-independent and culture-independent approach that enables the concurrent detection of RNA and DNA viruses, as well as viroids, even when present at very low titers in plant samples. In studies conducted in China and India, a novel *Illarvirus*, apple necrotic mosaic virus, was identified in apple trees displaying symptoms of mosaic disease, despite testing negative for apple mosaic virus (Noda et al., 2017; Nabi et al., 2022a,b). Additionally, sequencing confirmed the presence of apple rubbery wood virus 1, apple necrotic mosaic virus, citrus virus A, apple stem pitting virus, apple stem grooving virus, apple chlorotic leaf spot virus, and apple stem grooving virus-Shimla in pear samples (Khan et al., 2024a,b).

### 4.6 Clustered regularly palindromic/CRISPR-associated

Clustered regularly interspaced short palindromic repeats/CRISPR-associated (CRISPR/Cas) system has been developed into a potent tool in molecular biology and genetic engineering (Doudna and Charpentier, 2014). The remarkable target specificity of the CRISPR/Cas system, which allows precise gene editing by only changing the guide RNA (gRNA) sequence, is one of its most notable benefits. CRISPR/Cas12a field-based visual assay is used to detect numerous viruses affecting apples in China in the context of combating apple necrotic mosaic virus (ApNMV), apple stem pitting virus (ASPV), apple stem grooving virus (ASGV), apple chlorotic leaf spot virus (ACLSV) (Jiao et al., 2021).

## 5 Management

**Figure 1:**
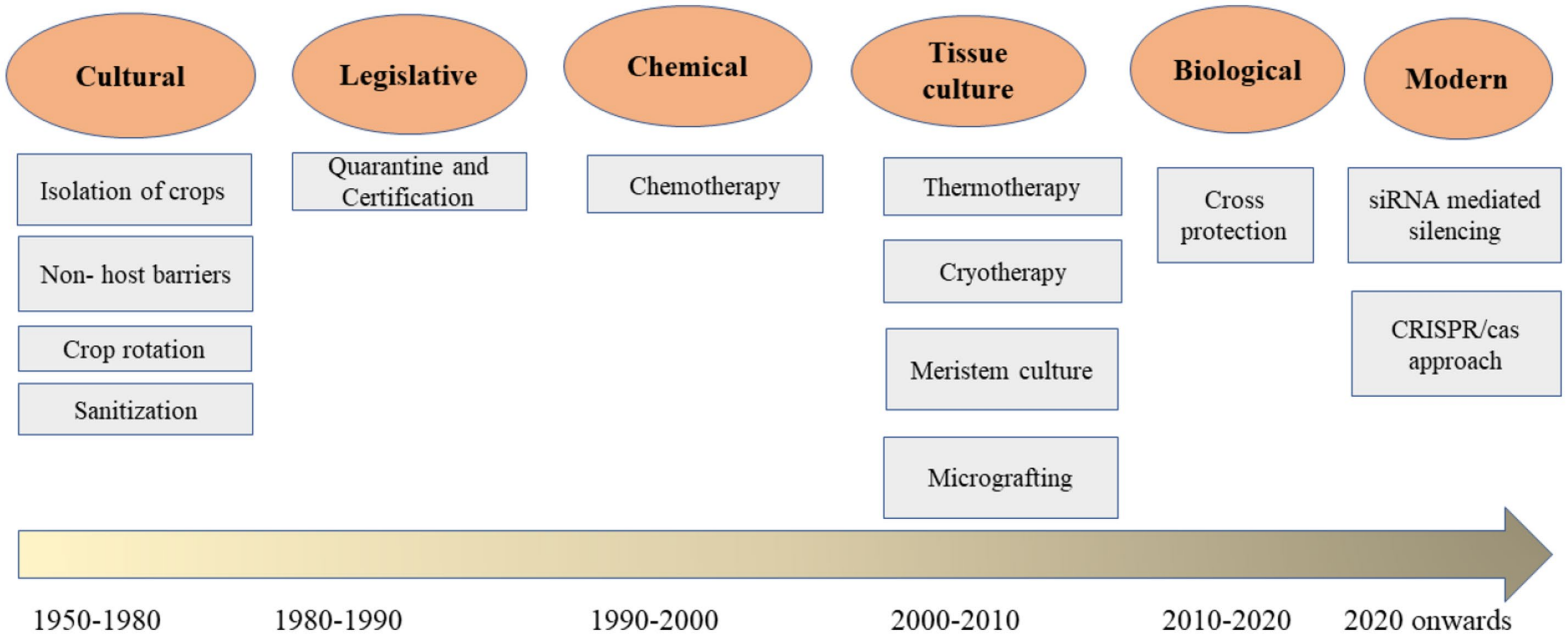
Schematic diagram shows various approaches to control pome viruses in horticulture.

To improve the management of viral diseases affecting apple, pear, and quince, rapid, efficient, and safe responses are essential for minimizing viral threats in global apple cultivation. Effective control of these viruses primarily relies on quarantine, isolation, sanitation, and certification programs, which depend on sensitive and specific detection and diagnostic methods. Biotechnological approaches, such as meristem tissue culture combined with thermotherapy and cryotherapy, are among the most effective strategies for obtaining virus-free plants and establishing virus-free plantations. Current management methods for apple, pear, and quince viruses include tissue culture, cross-protection, and chemical treatments. Emerging technologies, such as genome editing and RNA interference (RNAi), hold promise for improved management of viral diseases in these fruit crops. Quarantine regulations aim to prevent the introduction of pathogens into geographic regions where they have not been previously identified (Barba et al., 2015). These phytosanitary measures are governed by governmental authorities based on agreements established by international organizations. Currently, both mother plants and hardened tissue-cultured plants undergo quality and virus indexing assessments conducted at accredited test laboratories (ATLs) recognized by public and private institutions. This initiative exemplifies collaboration between the public and private sectors to produce and distribute certified tissue-cultured plants to farmers. The various management approaches are outlined below (Manzoor et al., 2023; Figure 1).

### 5.1 Meristem culture

Meristem culture is a crucial technique in tissue culture that involves isolating, sterilizing, and placing meristematic cells on culture media to develop complete plantlets. This method serves as an effective tool for eradicating viruses from infected plants. Virus elimination from infected cultivars can be achieved through various approaches, including thermotherapy, tissue culture, and cryotherapy, to establish pathogen-tested foundation sources for these cultivars. Currently, the most effective strategy for eliminating viruses from apple, pear, and quince plant material involves a combination of thermotherapy and shoot-tip grafting or meristem shoot-tip culture. For instance, thermotherapy at 38 ± 1°C has achieved an average elimination rate of 89.5% for ApNMV when applied to shoot tips (Hu et al., 2019). Additionally, a four-week high-temperature incubation (37°C) of apple plants infected with apple latent spherical virus (ALSV) has been shown to disable the movement of ALSV to new growing tissues (Yamagishi et al., 2016). The promotion of flowering via ALSV vectors, combined with virus elimination at elevated temperatures, facilitates accelerated breeding programs for apple and pear. Cold treatment has also proven effective in eliminating apple mosaic virus (ApMV) from apple planting material when tissues were treated with liquid nitrogen (Kaya, 2021).

### 5.2 Cross protection

A form of induced resistance in plants against viruses occurs when a prior infection with a virus protects the plant from subsequent infections by closely related viruses. This was tested on the Jonathan apple cultivar by infecting healthy trees with mild strains of ApMV. When these mildly infected trees were later exposed to moderate and severe strains of the virus, they showed resistance to further infections (Grimová et al., 2016).

### 5.3 Aptamers

Originating from the Latin word “aptus,” which means “to fit,” an aptamer is a brief, artificially designed oligonucleotide selected for its accurate binding properties with certain target molecules. To make aptamers for the two different coat proteins, MT32 and PSA-H, in the instance of the ASPV, a modified direct SELEX approach was used.MT32 and PSA-H have a high binding affinity for the MT32 aptamer, as evidenced by their estimated dissociation constants (KD) of around 55 and 83 nM, respectively. The remarkable specificity of these aptamers allows for the successful separation of ASPV from other viruses, including apple mosaic and apple chlorotic leaf spot viruses (Tungsirisurp et al., 2022).

### 5.4 Vitrification

The process for preserving axillary shoot tips (ASTs), which are 1 mm in length and contain 2–3 leaf primordial, involved several steps. The high success rates in eliminating ASGV and ASPV infections demonstrated the efficacy of the droplet-vitrification cryotherapy technique as a reliable method for producing ASGV- and ASPV-free propagating material in apple and quince (Souza et al., 2020).

### 5.5 Chemotherapy

To combat viral infections in apple shoots, a two-phase chemotherapy strategy was implemented. Initially, the shoots were immersed in a medium containing 20 mg/L of ribavirin for 4 weeks. Subsequently, in the second phase, the ribavirin concentration was escalated to 100 mg/L, with the treatment also extending for 4 weeks. This approach effectively curtailed the viral load to a significant degree. Following this treatment, shoot tips, which harbored 2–3 leaf primordia, were precisely excised and regenerated. Remarkably, the resulting plantlets exhibited a substantial reduction in viral infections. In the first phase, they demonstrated a 65% absence of ASGV and a 35% absence of ASPV. In the second phase, the efficacy of elimination improved further, achieving a 76% reduction in ACLSV (Paprštein and Sedlák, 2015).

### 5.6 Certification

Certification is an effective method to ensure that propagation material consistently meets two critical requirements: trueness to plant cultivar type and sanitary status. It is a collaborative process involving scientific and technical organizations, with multiple stakeholders contributing at different levels and holding distinct responsibilities. Certification protects both nurserymen, who supply vegetatively propagated plant material, and growers, who purchase these products. Several international organizations, such as North American Plant Protection Organization) (Mink, 1998) and EPPO (European and Mediterranean Plant Protection Organization (Roy, 2011), When developing a certification scheme, several key aspects are considered: (a) defining the steps involved in certification, (b) categorizing plant material at each stage of the scheme, (c) identifying the most harmful pathogens to be excluded from certified material, and (d) recommending the most reliable detection methods. Throughout all stages of certification, the multiplication history of the plants must be documented (Barba et al., 2015). In the case of pome fruits, certification programs control various viruses affecting apple, pear, and quince. Some of the major viruses managed through certification include apple mosaic virus (ApMV), apple chlorotic leaf spot virus (ACLSV), apple stem grooving virus (ASGV), and apple stem pitting virus (ASPV) in apples and pear blister canker viroid (PBCVd). These viruses can significantly impact fruit quality and tree health, making their exclusion through certification programs essential for sustainable orchard production (Eastwell, 2017; FontdevilaPareta, 2024; Chofong et al., 2024).

## 6 Conclusion and future prospects

A number of systemic pathogens, including viruses, have a negative effect on apple, pear and quince fruits, which causes the establishment of orchard diseases with broad repercussions. Managing diseases caused by viruses in apple orchards is essential for maintaining tree health, fruit quality and overall orchard sustainability. This requires a combination of control measures, accurate diagnosis, and international cooperation to prevent the spread of pathogens across borders. Accurately diagnosing the illnesses and their underlying causes is essential for the success of these management efforts. The key to treating viral infections in fruit trees is, in fact, diagnosis. In order to control viruses in apple, pear and quince trees or their propagation materials and to guarantee the longevity of agricultural practices, prompt viral identification is essential. In the future, managing and detecting apple, pear and quince viruses will rely on a combination of genetic engineering to create virus-resistant varieties, advanced molecular techniques for rapid detection, remote sensing and AI for monitoring orchards, data-driven approaches for outbreak prediction, and collaborative efforts among researchers and farmers. Additionally, user-friendly testing kits, mobile apps, and biosecurity measures will play crucial roles in preventing and managing virus outbreaks, ensuring the health and productivity of apple, pear and quince orchards in a changing agricultural landscape.
